# Trends in general practitioner dispensing of thickeners in the NHS England, 2019–24

**DOI:** 10.1371/journal.pone.0332357

**Published:** 2026-07-23

**Authors:** Arlene McCurtin, Lindsey Collins, Owen Doody, Dervla Kelly, Tracy Lazenby-Paterson, Dominika Lisiecka, Alison Smith, Shaun O’Keeffe, on behalf of the Swallow Perspectives, Advocacy & Research Collective

**Affiliations:** 1 School of Allied Health, University of Limerick, Limerick, Ireland; 2 Centre for Implementation Research (CIRcUL), University of Limerick, Limerick, Ireland; 3 Centre for Applied Dementia Studies, University of Bradford, Bradford, United Kingdom; 4 Department of Nursing & Midwifery, University of Limerick, Limerick, Ireland; 5 School of Medicine, University of Limerick, Limerick, Ireland; 6 Intellectual Disabilities Service, NHS Lothian, Edinburgh, United Kingdom; 7 Department of Nursing and Healthcare Sciences, Munster Technological University - Kerry Campus, Tralee, Co Kerry, Ireland; 8 NHS Hertfordshire and West Essex Integrated Care Board - 06N, Tring, Essex, United Kingdom; 9 Department of Geriatric Medicine, Galway University Hospital, Galway, Ireland; King Faisal University, SAUDI ARABIA

## Abstract

**Introduction:**

Oropharyngeal dysphagia is common and often results in aspiration. Thickeners are widely used to thicken liquids to slow the liquid bolus and prevent aspiration and aspiration pneumonia, despite multiple concerns about their use. To date, there is little data on prescribing/dispensing practices. This study aims to provide the first overview of thickener dispensing practices, serve as foundational material for parties interested in research, practice and policy making, and guide further work in this area.

**Method:**

We explored English general practitioner thickener dispensing practices for the five-year period 2019–2024 via the NHS Business Services Authority ePACT2 database and using descriptive statistics and linear regression to analyse the data. We hypothesized that with a large data set, it would be possible to detect trends in dispensing.

**Results:**

Between 2019 and 2024, nearly three million thickening items were dispensed in primary care in England at a cost of nearly 90 million sterling. Thickened liquids is primarily an intervention for older people although there was a significant decreasing trend in dispensed thickener items in adult populations. Thickener dispensing has increased in the youngest age group (0–1-years-old). The cost of thickeners to the NHS has risen in the last year under study despite the significant decline in use observed for most age groups. Of dispensed items over the five-year period, the vast majority were gum-based.

**Conclusion:**

While the data provided in this study is limited to GP dispensed thickeners in England, it provides researchers, clinicians and policy makers with initial information on dispensing practices. From the available data, a decreasing dispensing trend is seen in the context of rising costs, older people comprise the population who primarily receive thickeners and gum-based products dominate the market. This study raises a number of questions regarding the use of thickeners for people with swallowing problems.

## Introduction

Oropharyngeal dysphagia is common, with one in 17 adults affected [1]. The occurrence is particularly high in older people, specifically those in long-term residential care (LTRC), with a prevalence of 56% identified in a recent study [2]. Dysphagia is defined as a functional impairment that either prevents or limits the intake of food and fluids, and which makes swallowing unsafe, inefficient, uncomfortable or affects quality of life [3]. It increases the risk of penetration and/or aspiration of food and fluid into the airways. Health care professionals widely use thickeners to try and prevent aspiration by thickening liquids in order to increase viscosity and slow the flow of the bolus. Thickeners are ideally recommended after a clinical swallow exam, which is performed by a registered and dysphagia-trained Speech and Language Therapist (SLT), after which it is usually prescribed by the appropriate physician. While it may also be prescribed by nurse prescribers, the evidence for nurse prescribing of thickeners is currently limited [4]. SLTs have typically demonstrated high levels of use of thickeners [5,6] however, a drift towards decreased recommendations is suggested by the results of a recent international survey, which highlights that only 20% of SLTs frequently recommend the intervention [7].

The widespread use of thickeners for people with swallowing problems has come under increased scrutiny and criticism in recent years with poor evidence that they achieve a reduction in aspiration pneumonia [8] and a recent systematic review recommended against their use [9]. Potential adverse effects and unintended consequences include dehydration, urinary tract infections, thirst, oral and pharyngeal residue and impaired medication bioavailability [10]. Other issues include dislike of thickened liquids by people with swallowing problems, mainly because it impacts both their quality of life and enjoyment of drinking [11]. Informed decision-making deficits are also noted which impact treatment adherence [12,13]. The Royal College of Speech & Language Therapists (RCSLT) recently issued a position statement [10] and a subsequent paper [14] advising against thickeners being used “as a blanket approach or go-to treatment” and highlighted the need for decisions to be made ‘through a process of informed consent following a holistic assessment that includes consideration of the potential impact on health and quality of life’ (p4).

In the annual year 2023/24, thickeners were the 7^th^ highest spend category out of all (35) nutrition borderline substance categories in the UK [15]. The US market in thickeners was worth circa $584.6 million in 2023 and is projected to rise to $874.1 million in 2033 [16]. However, thickener dispensing has not yet been explored in the research literature and there is a lack of data available on prescribing or dispensing practices internationally to help researchers, clinicians and policy makers understand thickener dispensing and inform future planning and policy development regarding this widely-used and consequential intervention. It is also not known at present, for example, whether the current debate regarding the use of thickeners has impacted prescribing/dispensing practices. It is therefore timely that thickener dispensing is explored using the limited available data. In this study, we accessed the NHS Business Services Authority ePACT2 database [17] to examine dispensing trends over the last five years. While the public information regarding dispensing practices is currently limited to the NHS England GP dispensing, this data will provide the first overview of thickener dispensing practices, serve as foundational material for parties interested in research, practice and policy making, and guide further work in this area.

### Aims

To explore the data on English GP dispensing of thickeners for the period 2019–2024 and identify dispensing practices and trends.

## Method

### Data collection

Every NHS prescription issued and dispensed for a general practice patient is entered onto the administrative database of the NHS Business Service Authority (NHS BSA), the ePACT2 (electronic Prescribing Analysis and Cost) [17]. The ePACT2 database is publicly available and comprehensive in managing NHS primary care prescribing and dispensing costs across England. Dispensed items are those items for which prescriptions were written by general practitioners and subsequently filled. Data was obtained to conduct an analysis of thickener dispensing on ePACT2 data with retrospective analysis performed on the anonymised data for the five-year period 2019–2024. Inclusion criteria were limited and defined by the NHS BSA tabulated data and included: GP dispensing, primary care, thickeners, age ranges (0–1 years old, >1 year-17.11years old, >17 years – 64.11years old,>above 65 years old), type of product dispensed (gum, starch, carob) and cost per item changes within the period under study. Data on geographical location, clinical conditions etc. was not available. The authors received permission to explore and publish this data from the NHS BSA who provided the data on an Microsoft Excel (version 2511) spreadsheet for analysis in April-May 2025 [18].

### Data analysis

Data were analysed descriptively. The primary outcomes of interest for this report were the linear trends in total number of thickeners dispensed in the different age groups. These was analysed using a linear regression calculator (Statistics Kingdom) [19]. R-squared – the percentage of the variance explain by the regression – was used to measure the goodness-of-fit of the model. The F test was used to check if the entire regression model is statistically significant. The Annual Percent Change (APC) and associated 95% confidence Interval (CI) was calculated using the exponents of the slope coefficients from linear regression of the natural logs of the dispensed item values as the dependent variable and the year as the independent variable.

## Results

Between 2019 and 2024, nearly three million thickening items were dispensed in primary care at a cost of nearly 90 million sterling (Table 1). The majority of dispensed items were for the 65+ years old age group.

**Table 1:** No. of thickening items dispensed based on GP prescriptions and age in England 2019−24.

Age Group	Year	No items dispensed	Total Cost
0–1-year-olds	19-20	28,997	191,111.77
	20-21	43,807	271,256.59
	21-22	44,889	281,435.73
	22-23	45,914	300,395.20
	23-24	49,397	354,033.84
**Subtotal**	**19-24**	***213,004***	***1,398,233.13***
>1–17.11-year-olds	19-20	44,070	914,565.87
	20-21	42,071	933,036.69
	21-22	43,577	974,256.99
	22-23	44,676	1,027,913.94
	23-24	43,737	2,088,785.20
**Subtotal**	**19-24**	***218,131***	***5,938,558.69***
18–64.11-years-olds	19-20	89,708	4,077,456.82
	20-21	88,171	4,126,461.78
	21-22	84,455	3,938,929.16
	22-23	82,030	3,764,450.29
	23-24	79,230	3,743,609.40
**Subtotal**	**19-24**	***432,264***	***19,650,907.46***
65+ years olds	19-20	449,293	14,357,480.70
	20-21	419,857	13,141,608.53
	21-22	396,057	12,038,886.02
	22-23	391,591	11,488,936.30
	23-24	380,118	11,503,136.32
**Subtotal**	**19-24**	***2,036,916***	***62,530,047.87***
**Total**	**19-24**	**2,900,315**	**89,517,747.15**

**Fig 1:**
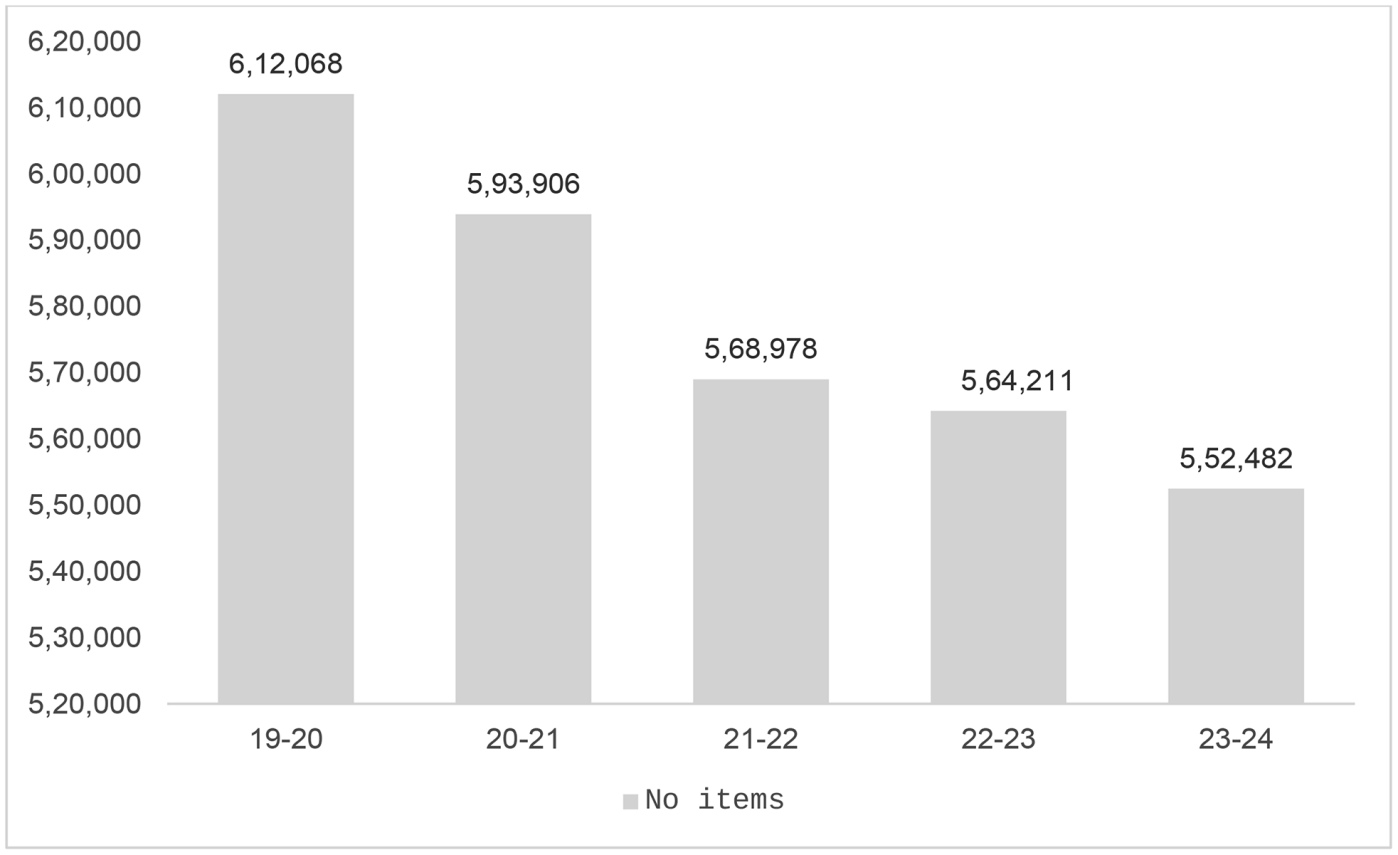
Total no of thickening items dispensed 2019−24.

Approximately 612,000 items were dispensed in 2019–2020 and 552,482 were dispensed in 2023–2024, a 15% drop in dispensed items in the five-year period (Fig 1). A reduction in the total number of dispensed items is evident year on year.

In the 0-to-1-year age group, which accounts for the least dispensed items, there was a 70.4% increase overall in these 5 years (Table 1). The linear regression equation for X (year) predicting Y (number of items) was Y = −8627490 + 4290X. R square was 0.74 (an R square of greater than 0.9 is regarded as high and ‘good’) showing that 74% of the variation in dispensed items was explained by the year; adjusted R square was 0.66. The standardised beta coefficient, which measures how many standard deviations the dependent variable (Y) changes for a one-standard-deviation change in the independent variable (X), was 0.86 (95% CI −0.09 to 0.99). Overall, the linear model showed a non-significant increasing trend: F(1,3) = 8.52, p < .063. The APC (95% CI) was + 11.6% (−2.3 to +27.9%).

In the 1-to-17.11-year age group, there was a negligible 0.7% increase in thickened items dispensed between 2019−20 and 2023−24 (Table 1). The linear regression equation was Y = −376553.7 + 207.9X. R square was 0.12; adjusted R squared was −0.17. The standardised beta coefficient was 0.31 ((5% CI −0.79 to 0.94). There was no significant linear trend: F(1,3) = 0.4, p < .58. The APC was 0.5% (95% CI −2.0 to +2.9%).

In the 18-to-64.11-year age group, there was an 11.7% decrease in thickened items dispensed between 2019−20 and 2023−24 (Table 1). The linear regression equation was Y = 5561022.5 − 2709.7X. R square was 0.99, as was adjusted R squared, showing that the year explained almost all variation in dispensed items. The standardised beta coefficient was 0.99 (05% CI 0.92 to 1.0). The declining linear trend over 5 years was highly significant: F(1,3) = 271.53, p < .001. The ACP was −3.3% (95% CI −2.6 to −3.9%).

In those aged 65 years or more, there was a 15.4% decrease in thickened items dispensed between 2019−20 and 2023−24 (Table 1). The linear regression equation was Y = 457368 − 16661.6X. R square was 0.92 and the adjusted R squared 0.82. The standardised beta coefficient was 0.95 (95% CI 0.48 to 1.0). This declining linear trend over 5 years was again significant F(1,3) = 32.41 (p < 0.012).). The ACP was −4.1% (95% CI −2.0 to −6.3%).

**Fig 2:**
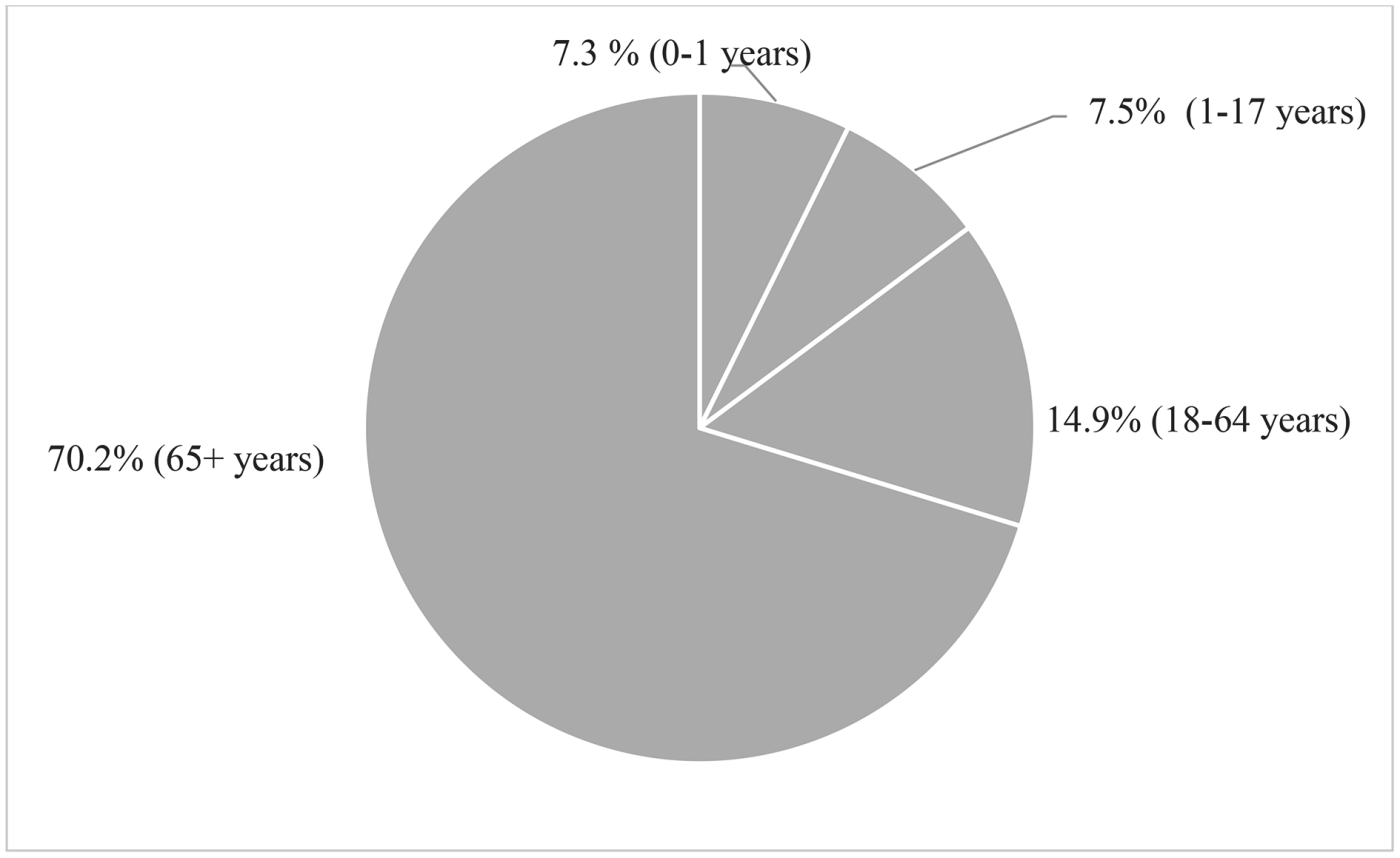
Percentage of total thickening items dispensed by age between 2019-2024.

The older age group (65+ years old) accounts for most dispensed items, with more than two million thickening items (70.2%) being dispensed in primary care for older people at a cost of more than 62 million sterling (Fig 2).

**Fig 3:**
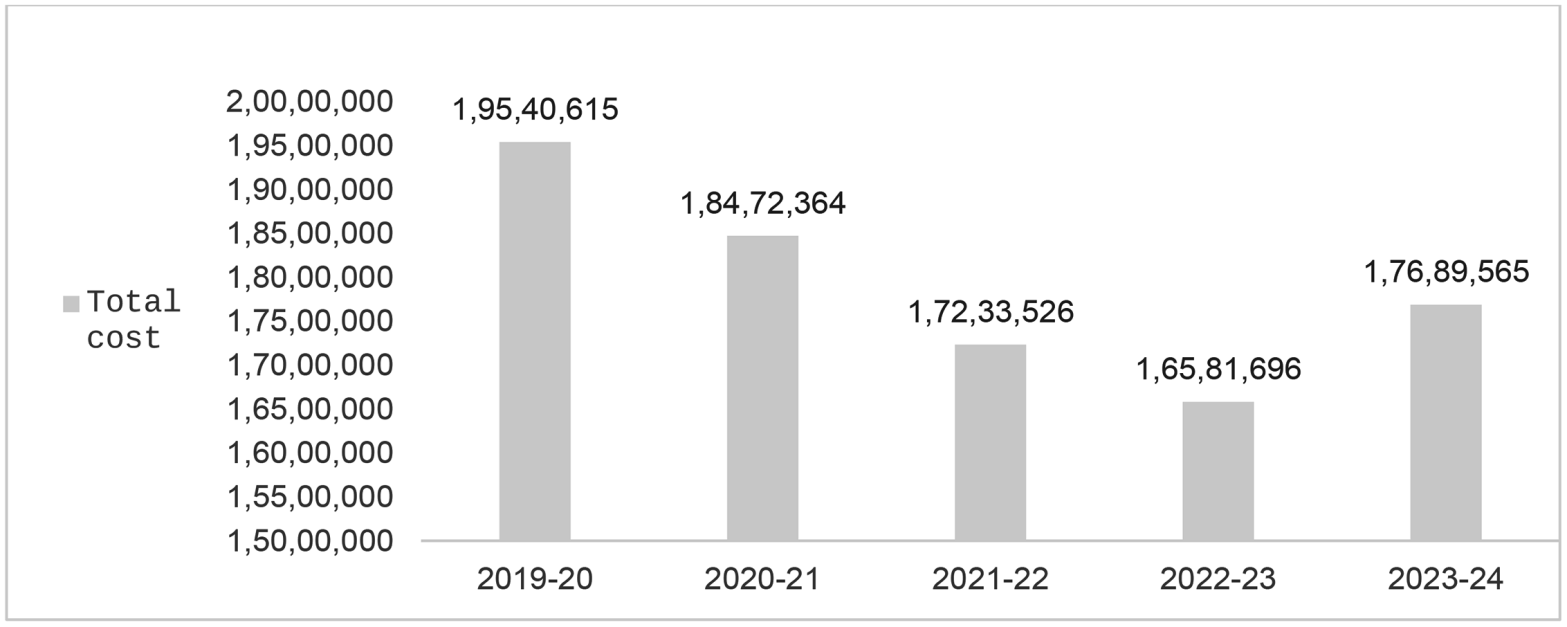
Total cost of thickening items dispensed 2019−24.

Despite a reduction in the total number of dispensed items and a decreasing trend from 2019−2023, costs are seen to rise in the most recent year for which data is available (Fig 3). Although nearly 12,000 less items were dispensed in the 2023–2024 period across all age groups, constituting a 15% drop, dispensed items cost the NHS nearly 7% more in the 2023−24 period than for the previous year.

Price increases vary between the selected products ranging from 6.6% to 36.8% (Table 2). Carobel products have shown the greatest price increase.

**Table 2:** Price changes per dispensed thickening items for main dispensed products.

Company/product	Price pre-2020	Price as of 01/04/2024	% increase
Thick & Easy	5.41	6.10	12.75%
Resource Thicken Up	8.46	9.02	6.6%
Nutilis	5.50	6.75	22.7%
Nutilis Clear Sachets	11.04	14.0	26.8%
Carobel	2.91	3.98	36.8%

Of dispensed items over the five-year period, approximately 77% were gum-based, 12% were starch-based and 10% were carob-based (Table 3). The cost of gum-based thickeners was higher than other products representing 87% of total costs (Table 3).

**Table 3:** Type of thickening items dispensed based on age 2019−24.

Age Group	Year	No. gum-based items	Gum-based costs	No. carob-based items	Carob-based costs	No. starch-based items	Starch-based costs
0–1year-olds	19-20	338	2,216.56	28,528	184,235.96	131	4,215.52
	20-21	465	5,131.10	42,881	264,693.08	461	4,852.91
	21-22	145	2,060.08	44,502	276,659.37	242	2,716.28
	22-23	91	1,305.85	45,689	297,312.78	134	1,776.57
	23-24	98	1,202.80	49,204	351,611.02	95	1,220.01
**Subtotal**	**19-24**	***1,137***	***11,916.39***	***210,804***	***1,374,512.21***	***1,063***	***14,781.29***
***% of total***	**19-24**	***0.5***	***0.85***	***99.1***	***98.7***	***0.5***	***1.06***
1–17.11year-olds	19-20	16,946	525,823.09	16,981	169,630.30	10,143	219,112.48
	20-21	17,405	571,341.27	16,910	177,878.88	7,756	183,816.54
	21-22	19,523	627,810.44	17,032	176,477.55	7,022	169,969.00
	22-23	21,201	688,927.53	17,699	196,594.51	5,776	142,391.90
	23-24	22,518	777,858.86	17,481	213,274.61	3,738	1,097,651.73
**Subtotal**	**19-24**	***97,593***	***3,191,761.19***	***86,103***	***933,855.85***	***34,435***	***1,812,941.65***
***% of total***	**19-24**	***44.7***	***53.7***	***39.5***	***15.7***	***15.8***	***30.5***
18–64.11years-olds	19-20	64,802	3,217,863.50	225	5,280.67	24,681	854,312.65
	20-21	70,086	3,481,938.72	248	5,165.95	26,503	639,357.11
	21-22	69,986	3,418,916.09	218	4,874.16	14,251	515,138.91
	22-23	70,095	3,307,791.45	216	4,760.79	11,723	451,898.05
	23-24	71,432	3,394,703.91	229	5,527.79	7,569	343,377.71
**Subtotal**	**19-24**	***346,401***	***16,821,213.67***	***1,136***	***25,609.36***	***84,727***	***2,804,084.43***
***% of total***	**19-24**	***80.1***	***85.6***	***0.3***	***0.13***	***19.6***	***14.27***
65 + years olds	19-20	358,467	12,375,588.54	66	395.61	90,760	1,981,496.55
	20-21	362,596	11,963,560.48	97	487.21	57,164	1,177,560.84
	21-22	355,184	11,212,639.18	95	590.72	40,778	825,656.12
	22-23	360,522	10,868,291.91	90	461.58	30,979	620,182.81
	23-24	364,007	11,154,580.00	100	1,685.77	16,011	343,922.37
**Subtotal**	**19-24**	***1,800,776***	***57,574,660.11***	***448***	***3,620.89***	***235,692***	***4,948,818.69***
***% of total***	**19-24**	***88.4***	***92.08***	***0.02***	***0.01***	***11.57***	***7.9***
**Total**	**19-24**	**2,245,907**	**77,599,551.36**	**298,491**	**2,337,598.31**	**355,917**	**9,580,626.06**
**% of total**	**19-24**	***77.44***	***86.7***	***10.3***	***2.6***	***12.3***	***10.7***

**Fig 4:**
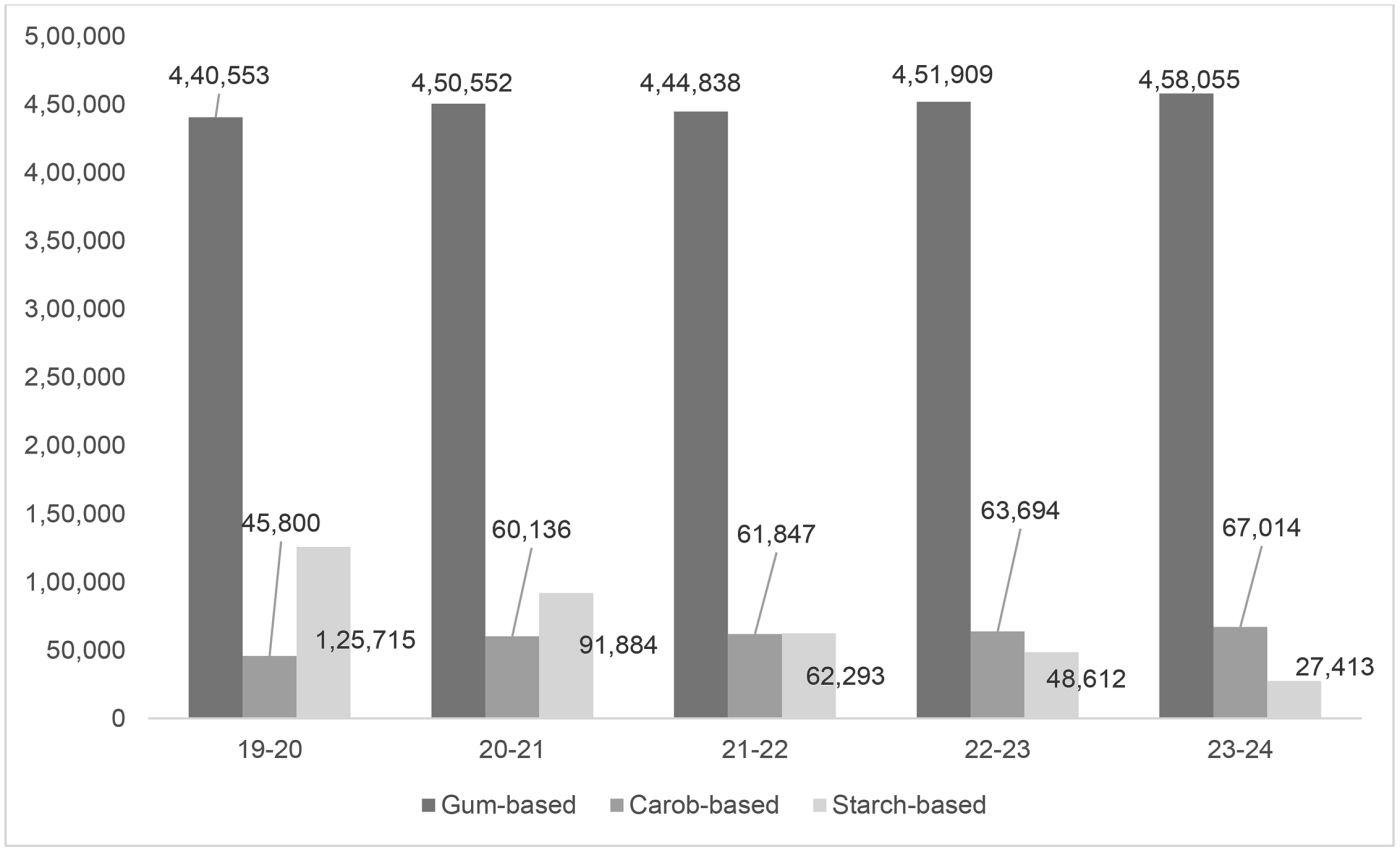
No. of thickening items dispensed by type 2019−24.

Gum-based thickeners dominate dispensing (Fig 4). Dispensing of starch-based items decreased. Carob-based dispensed items increased by approximately 68% over the period showing a large growth in dispensed items. This, combined with increased cost of Carobel products can be said to be a contributor to overall increased costs. Dispensing of gum-based items increased fractionally by nearly 3% while starch-based dispensed items decreased significantly by approximately 78%.

**Fig 5:**
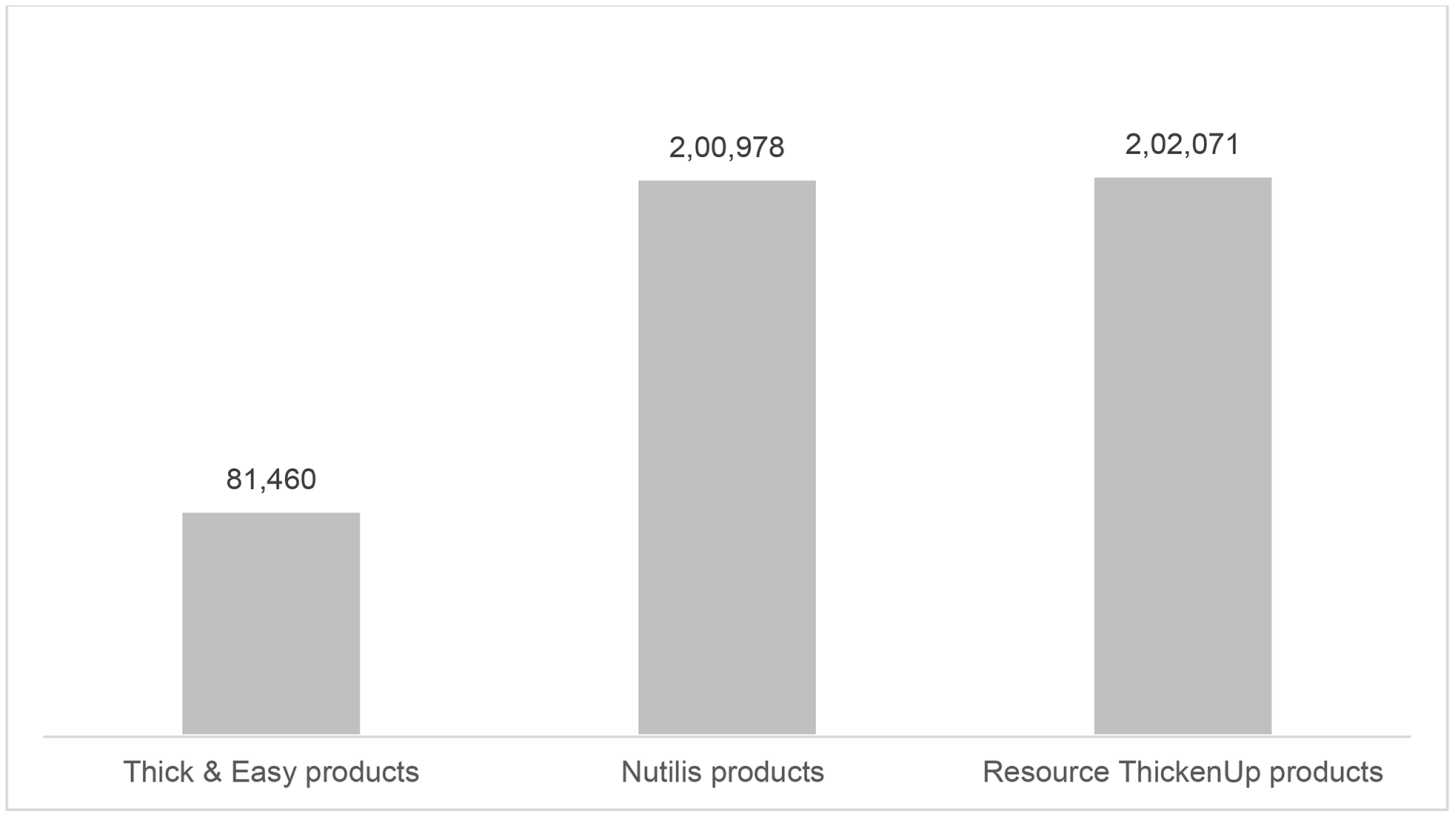
No of total items dispensed by primary suppliers 2023−24.

While a range of 33 items were dispensed from a range of companies during the last year for which data is available, three companies supply the majority of dispensed items (Fig 5). Resource Thicken Up and Nutilis products represent the primary dispensed items in 2023–2024, accounting for 73% of dispensed products in that year.

## Discussion

Much has been written about thickeners over the last number of years, resulting in an RCSLT position paper on the topic [14] and the funding of a National Institutes of Health Research (NIHR) grant focused on de-implementation of the thickened liquid intervention. To date however, there has been a deficit of information regarding thickener prescribing/dispensing practices, and this paper is intended to help rectify this situation.

### Number/cost of items dispensed

Our results show a clear and significant decline in the number of thickening items dispensed in primary care between 2019 and 2024. It is important to note that this data reflects only GP dispensing and only in primary care, which is likely to be an underestimation of overall thickener use, as the data does not account for dispensing in other community settings such as long-term residential care facilities or in acute or rehabilitation settings. While we only examined five years of data and this meant the a priori power of our study to detect a significant change was low, the decline in GP dispensing was consistent and of such a magnitude, that our results are statistically significant. As a result of this trend in declining prescriptions, the cost of thickeners to the NHS has reduced, although not as much as might be expected. This can be explained by data from the last year under study (2024), which highlights a recent increase in overall dispensing costs due to significantly increased unit costs for all products. As a result, costs have risen by approximately 6.7% between 2023–2024, when they had seen a decline of just over 15% in the previous four-year period.

The data cannot explain why such a decline has occurred in England, however, the first of the ‘backlash’ papers on the topic in major journals were published from 2016 onwards [20–22] and have continued since. Awareness of the ongoing debate including adherence issues and treatment burden [23,24] and (appropriately, in our view) a greater caution among SLTs in recommending and triggering prescriptions of thickeners seems a plausible explanation. One significant clinical governance development in recent years in England and elsewhere has been the increase in “risk feeding” policies such as ‘Eating and Drinking with Acknowledged Risks’ (EDAR), which aim to reduce unnecessary use of modified diets in people with dysphagia [25]. Although this is an admirable goal, the concept and approach have been criticised [26]. It is possible, although – given the lack of impact seen in studies to date [27] – unlikely, that this too has influenced the downward trend in prescribing of thickeners.

Another possible reason for the observed decline may have its origins in SLT staff shortages with 19% of SLT posts in England vacant in the spring of 2024 [28]. The older population in England, the major recipient of dispensed thickeners, continues to rise rather than fall, and there has been no major decline in the prevalence of the common conditions that cause dysphagia. Hence these would not explain our findings. The years analysed included the years when the COVID pandemic was active and it is possible that there was reduced assessment and identification of dysphagia during this period, or that the pandemic led to the premature death of vulnerable older people with neurodegenerative conditions. However, this is unlikely to explain the ongoing decline in dispensing (and long COVID is a potential cause of dysphagia). The cost-of-living crisis may also be a contributory factor, especially given the NHS England decision to move many over the counter medicines off prescriptions. Most people under 60 now have to pay for prescriptions in England, and some may decide not to fill some of their prescriptions because of cost [29].

Of note, SLTs are not the only health profession recommending use of thickeners. There is limited evidence however, that nurse prescribers for example, are prescribing thickeners [4] and physician prescriptions continue to dominate prescribing in this area. Nevertheless, there is international evidence that thickeners are being used without the benefit of a clinical swallow exam being carried out by specialist dysphagia-trained SLTs. This includes Ireland, where 70% of nurses reported that thickeners and modified texture diets were often started in the absence of a swallow exam performed by an SLT [30]; the USA where 80% of US SLTs encountered nursing-initiated texture modified practices [31]; New Zealand, where one third of residents (n = 35,460) in long-term residential facilities were given texture modified diets without the benefit of an SLT consult or exam [32], and; Canada, with health care assistants rarely accessing swallowing evaluations and demonstrating an overreliance on texture modified diets [33]. Estupinan Artiles et al.’s (2024) recent survey of Irish long-term residential settings highlighted that residents were provided with texture modifications, including thickeners, if they had suspected swallowing problems [34], while Bice et al.’s (2023) US-based study, provides evidence that two thirds of those receiving a modified solid/liquid diet in a continuing care setting (n = 120), did not actually have an eating drinking and swallowing problem [35]. Thus, while a decline is noticed in GP dispensing, and the reduction in dispensed thickener items is a positive finding in our view given the lack of evidence of benefit and the potential adverse effects, this trend may not reflect some practices in long-term residential settings, raising questions regarding overdiagnosis, overtreatment and treatment burden even in the absence of the condition for which the intervention is designed, and probably by individuals not trained in dysphagia. Evidence suggests that around one in 20 patients are exposed to preventable harm in medical care with incidents related to drugs and other treatments accounting for the largest proportion of preventable patient harm [36].The WHO estimates that 50% of prescribed and marketed medications are unsuitable, and 50% of patients administer these medications improperly, emphasizing the need to address medication iatrogenesis risk [37] and reinforcing the importance of continuously re-evaluating clinical practices like thickener use in light of evolving research evidence and patient safety outcomes. Further, in the UK, food and drink thickeners are not tightly regulated as they are considered nutritional rather than medical products, which means they are subject to general food safety regulations rather than specific standards related to medicines

### Age of people receiving dispensed items

The vast majority of community dispensed items – approximately 70% - were provided to older people and thickened liquids can legitimately be considered as an intervention primarily targeting older people. The data provided may be an underestimation given the LTRC practices cited above which may bypass SLT swallow exams and physician prescribing. Hammer-Castellanos et al. (2024) for example, in a survey of 253 US nursing homes, found a mean of 8.3% of residents were receiving the thickeners, with a high of 28% in some facilities [38]. Irrespective, the high use of thickeners for this age group raises questions regarding the vulnerability of older people for this poorly evidenced, disliked and consequential intervention in both the community and LTRCFs, where the implications for nutritional status and hydration monitoring is significant [10] and where thickener use often intersects with broader geriatric care policies. Further, McGrail and Kelchner (2015) have shown that cognitive deficits predict oral fluid intake in people post-stroke receiving thickened liquids, raising valid questions regarding the use of thickeners in people with cognitive deficits [39]. There is good reason to believe that informed consent for use of thickeners is either not sought or may be based on a misrepresentation of the evidence. The dominance in thickener dispensing for the oldest age group is also interesting when considering epidemiological information. In England for example, 19% of the population is above 65+ years [40] and thus constitutes a small percentage of the population, but with a higher thickener dispensing rate.

Data on the youngest age group (0–1-years old) and in contrast to other age groups, highlights a significant rise in dispensed items over the five-year period, concurring with reports of witnessing a surge of thickener use with this population [41]. While the reason for this particular trend is unclear, in recent years, more SLT posts in neonatal facilities have been created which may have resulted in an increased focus on swallowing problems. More likely however, is that thickener use in this population is not limited to the treatment of swallowing problems but used for conditions such as gastro-oesophageal reflux disease (GORD) to reduce the number of regurgitation episodes [42]. This practice reflects guidelines which recommend thickening as the first-line approach to treat GORD in infants and young children [43]. Thus, the increasing trend of dispensed thickened items for the youngest age group, may have its roots not so much in swallowing problems, but in other medical conditions.

### Type of thickeners dispensed

The vast majority of thickeners dispensed were gum-based. Starch-based thickeners have significantly decreased as a proportion of dispensed items over the five-year period studied. According to the Specialist Pharmacy Service [44], while starch-based thickening agents were widely used in the 1990s, gum-based thickening agents have gained popularity more recently. This is supported by the dispensing data. In contrast to starch-based thickeners, xanthan gum-based thickened fluids -in the UK, gum-based thickening agents are mostly xanthan gum-based- are reported to be effective in reducing aspiration for several reasons, including not increasing pharyngeal residue, better texture perception and better resistance to salivary amylase [45]. Calmarza-Chueca et al. (2022) also report better stability across time and temperature (6.5% vs. 43%) [46], although Kim and Yoo (2018) have shown gum-based beverages thickening after a 15–45-minute prep window [47]. This is important as it is not uncommon, particularly with elderly people, for a thickened drink to be sitting for several hours. Further, Kim et al. (2017) showed that gum-based thickeners significantly altered sensory attributes [48]. Thus, questions remain on the quality and performance of gum-based thickeners despite their dominance of the market.

Carob-based dispensed items showed a large relative growth during this five-year period and carob seed/gum is used primarily for the youngest age group. It is reported to be a high viscosity product which has the property of absorbing water up to 40%, making it an effective thickener, stabilizer and emulsifier [49]. Carobel is the only ACBS approved thickener for children under 3 years old, with limited options being available due to the general high sodium content in thickeners. In this population, it is licensed for reflux and regurgitation rather than dysphagia. Once children reach the age of 3 years, it is recommended that they switch to gum-based thickeners [50].

## Conclusions

While the data provided in this study is limited to GP dispensed thickeners in England, it provides researchers, clinicians and policy makers with initial information on dispensing practices. From the data it is evident that there is a significant decreasing trend in dispensed thickening items in adult populations and that the use of thickeners is primarily an intervention for older people. Thickener dispensing has increased in the youngest age group (0–1-years-old). The cost of thickeners to the NHS has risen in the last year in particular despite the significant decline in use observed for most age groups. From the available data, thickeners can be said to be primarily an intervention for older people and gum-based products dominate the market. This study raises a number of questions regarding the use of thickeners for people with swallowing problems.

## Limitations

The nature of the database used in the study means our data are highly reliable. However, there are limitations in the data also. It represents a small sample size – GP dispensing in community care in England – thus generalisations to other areas of practice, practitioners and geographical locations cannot be inferred. It represents dispensed products only; more prescriptions may have been written but not dispensed, for example, if the person chose not to take thickener. We cannot know whether dispensing was occurring in residential care within the community or in the community itself, or if dispensing resulted from SLT recommendations. We do not know if differences exist based on geography. It would also be helpful to have data from earlier years to better judge whether the trajectory of decline can be attributed to the debate about thickened liquids. Our data are also too early to show any effect from the RCSLT publications, and it will be interesting to see whether the decline continues. Further, due to the nature of the data retrieved, there is limited information on outcomes related to use of dispensed thickeners, for example, a reduction in aspiration events and hydration status and whether the individuals prescribed thickeners actually used the product – there is evidence that many individuals do not [50].

## Recommendations

1. A number of findings require further exploration, including the dominant use of thickeners for older people and the increase in use of thickeners for the youngest group, both of whom are particularly vulnerable groups.
2. It would be helpful to explore data from earlier years not included in this study, to better judge whether the trajectory of decline can be attributed to the debate about thickeners. It will also be helpful to repeat the review of the data in the coming years to identify any effect from newer RCSLT policy, and it will be interesting to see whether the decline continues.
3. The development of multiple accessible prescription/dispensing databases is required for improved knowledge and to support decision-making and policy development. This includes, but is not limited to, geographical information, clinical conditions, outcomes, prescriptions written vs. dispensed items, prescribing by non-GP physicians and sources of thickener recommendations. There is also a lack of data on how often prescriptions for thickeners are reviewed, and while the requirements are likely to change over time, this is not well documented and can result in under-ordered or stock piling and waste [51,52].
4. Although there is an increased awareness among speech and language therapists regarding the debates surrounding thickeners, it seems likely that doctors and nurses will be less aware that the benefits of thickeners have been called into question. Education about thickeners targeting physicians, nurses, health-care assistants and care homes needs to be rolled out as a priority to establish an evidence-based and patient informed thickener culture, to improve safety and the care experience and to reduce overuse.
5. Continued research on gum-based thickeners is required given their dominance in dispensing practices including, but not limited to, physiochemical properties, safety and efficacy.
